# Decreased inhibitory control after partial sleep deprivation in individuals reporting binge eating: preliminary findings

**DOI:** 10.7717/peerj.9252

**Published:** 2020-05-29

**Authors:** Silvia Cerolini, Andrea Ballesio, Fabio Ferlazzo, Fabio Lucidi, Caterina Lombardo

**Affiliations:** 1 Department of Social and Developmental Psychology, Sapienza University of Rome, Rome, Italy; 2 Department of Psychology, Sapienza University of Rome, Rome, Italy

**Keywords:** Sleep deprivation, Binge eating, Executive functions, Inhibitory control, Disordered eating, Sleep

## Abstract

**Background:**

Poor executive functions are associated with dysregulated eating and greater caloric intake in healthy samples. In parallel, findings suggested that sleep deprivation impairs executive functions.

**Methods:**

We investigated whether partial sleep deprivation impairs executive functions in individuals reporting binge eating (BE, *N* = 14) and healthy controls (C, *N* = 13). Switch cost and backward inhibition were measured using the Task Switching Paradigm after a habitual night of sleep and after a night of partial sleep deprivation.

**Results:**

Results showed a Night by Group interaction on the backward inhibition. The two groups differed in the habitual night, evidencing higher inhibitory control in BE compared to C. Additionally, after partial sleep deprivation, compared to the habitual night, backward inhibition decreased in BE group. This preliminary study was the first to explore the impact of sleep deprivation on executive functions in participants reporting binge eating and healthy controls, thus highlighting their potential role in influencing eating behavior.

## Introduction

Executive functions (EFs) include inhibitory capacities and switching attention processes (Diamond, 2013). Emerging evidence suggests that EFs are underlying factors of eating behavior in healthy individuals. Specifically, poor EFs have been linked to greater caloric intake in both healthy children and adults (Lundahl & Nelson, 2015). Results on adults demonstrated that elevated impulsivity is associated with higher caloric consumption, especially when the reinforcing value of the food is high (Rollins, Dearing & Epstein, 2010). Moreover, poor inhibitory control has been associated with greater consumption of snacks and high-fat foods (Hall, 2012). Despite this first evidence supporting the cross-sectional relation between EFs and dietary intake, the lack of longitudinal studies does not allow to make solid conclusions about directionality (Egbert et al., 2019). Although most of the studies consider EFs as a factor that may influence dietary intake, there are also few pieces of evidence demonstrating the inverse relationship such as dietary intake influencing EFs (Egbert et al., 2019; Dohle, Diel & Hofmann, 2018). Considering this perspective, recent metanalytic evaluations found that obese participants showed broad impairments on EFs as inhibitory control, cognitive flexibility, working memory, decision-making, verbal fluency, and planning compared to normal-weight participants (Yang et al., 2018) and that weight loss intervention may improve EFs among overweight and obese people (Veronese et al., 2017).

Impairments in EFs have been shown to be also associated with eating disorders such as Bulimia and Anorexia Nervosa (Hirst et al., 2017). Nevertheless, it is still poorly understood whether poor EFs play a role in Binge Eating Disorder (BED) (Van den Eynde et al., 2011).

A systematic review by Lavagnino et al. (2016) suggested that inhibitory control was significantly impaired in obese individuals compared to normal-weight individuals; however, the presence of BED in obese individuals was not associated with inhibitory performance.

More recently, a study by Kittel, Schmidt & Hilbert (2017) investigating EFs in adolescents with and without BED and obesity, found that individuals with BED and obesity displayed significantly poorer inhibitory control compared to normal-weight individuals. Previous evidence, however, failed to find any difference in inhibitory control between obese individuals with and without BED (e.g., Galioto et al., 2012). Manasse et al. (2015) found that albeit individuals with binge eating displayed poorer EFs performance in problem-solving and inhibitory control, the difference in the latter was no longer significant after controlling for depressive symptoms. Also, Kollei et al. (2018) reported that individuals with BED and obesity showed poorer performance in reversal learning and decision-making tasks than individuals with obesity without BED. It is also worth noting that besides contrasting empirical results, it is still unclear how exactly the EFs affect BED.

In parallel, experimental findings suggested that sleep deprivation modulates EFs in healthy samples, although findings are not entirely consistent. Couyoumdjian et al. (2010) and Gorgoni et al. (2014) found that total sleep deprivation depletes inhibitory capacities, whilst a recent study from our group suggested that partial sleep deprivation might have a slight beneficial effect on non-inhibitory switching capacities as assessed in task switching paradigm (see below), but no effects on inhibitory capacities (Ballesio et al., 2018). Taken together, these findings may suggest that one mechanism by which disrupted sleep patterns may increase food intake is via changes of EFs (Lundahl & Nelson, 2015). According to Dohle et al.’s review (2018), EFs may also be considered as a mediator of the self-regulation of eating behavior, influenced by other variables. We hypothesize that sleep impairment may influence EFs, thus altering eating behavior. Additionally, a large body of literature supports the cross-sectional and longitudinal link between sleep impairments and increased food intake and obesity risk among children, adults, and older adults (Fatima, Doi & Mamun, 2015; Bacaro et al., 2020; Norton et al., 2018).

Unfortunately, empirical studies examining the effects of sleep deprivation on EFs in relation to eating behavior are lacking. A notable exception is a study by Cedernaes et al. (2014), reporting that total sleep deprivation impaired inhibitory control in response to food stimuli in healthy young men, increasing impulsivity in response to such food cues. Nevertheless, to the best of our knowledge, no study to date has examined the effects of sleep deprivation on EFs in binge eating. We aimed to fill in this gap by preliminarily evaluating whether partial sleep deprivation impairs EFs in individuals reporting binge eating and healthy controls. In contrast to Cedernaes and colleagues, we decided to use partial sleep deprivation (instead of total sleep deprivation) because of the following reasons: (a) most studies on the relationship between sleep and eating behavior or obesity use a partial sleep deprivation paradigm; (b) the partial sleep deprivation paradigm is closer to what usually happens in life. In fact, many people in western countries voluntarily curtail their sleep due, for instance, to the use of media or disordered sleep habits.

## Materials & Methods

### Participants selection

Data were collected within a more extensive study on the effects of partial sleep deprivation on eating-related cognitive and emotional variables, as previously described in Cerolini, Rodgers & Lombardo (2018). Specifically, twenty-eight participants (Mage = 23.75  ± 4.03, 21% male) were recruited among the student community of Sapienza University of Rome. After an initial online screening, during which participants were asked to complete self-report questionnaires and demographic information, eligible participants were contacted to take part in the study. Two groups were formed based on the total scores of the Binge Eating Scale (BES, Gormally et al., 1982) and the Disordered Eating Questionnaire (DEQ, Lombardo et al., 2004): the Binge Eating Group (BES ≥ 17, *N* = 14) and the Control Group (BES < 17, DEQ < 30, *N* = 14), and they were also matched for age and gender. BES is a 16-item questionnaire assessing the presence of certain binge eating behaviors such as controlling impulses towards food, feeling guilty after having eaten too much, having intrusive thoughts about eating, overeating in secret, etc. BES score of 17 is considered the clinical cut-off for the presence of binge eating. DEQ is a 24-items questionnaire assessing dysfunctional eating behavior-related patterns, such as restrictive eating. This scale includes items regarding reducing food intake to lose or maintain weight, ruminating and worrying about weight and body shape, engaging in intense physical exercise to lose weight, etc. The clinical cut-off score is 30. All participants voluntarily agreed to participate in the study and provided written informed consent. The consent form contained the explanation of the procedure, the list of potential risks and benefits participating in the study, and a disclaimer for the delivery of the equipment. In order to guarantee participants’ privacy, an alphanumeric code was created by each subject. The study has been carried out in accordance with The Code of Ethics of the World Medical Association (Declaration of Helsinki), and the procedure was approved by the Institutional Review Board of the Department of Psychology at the Sapienza University of Rome.

### Other materials

Insomnia and depressive symptoms during the preceding two weeks were measured using: the Insomnia Severity Index (ISI, Bastien, Vallières & Morin, 2001), a 7-items in which the score ≤ 7 is considered the cut-off for the absence of clinically significant insomnia; the Beck Depression Inventory (BDI-II, Beck, Steer & Brown, 1996), a 21-items in which total scores of 0–13 are considered indicative of minimal-range depression, 14–19 as mild, 20–28 as moderate, and 29–63 as severe.

#### Sleep assessment

Throughout the entire duration of the study, sleep was measured both subjectively, using sleep diaries, and objectively, through a portable electronic device called Zeo (Inc., Newton, MA) (Cellini et al., 2015), which was used to monitor the compliance to the sleep deprivation instructions as previously reported (Lombardo et al., 2020; Ballesio et al., 2018).

#### Executive function assessment

Participants completed the Task Switching paradigm (Jersild, 1927; Sdoia & Ferlazzo, 2008), to assess switching attention and backward inhibition. Data were collected as previously described by our group (Ballesio et al., 2018; Ballesio, Ottaviani & Lombardo, 2019; Ballesio et al., in press).

Participants were trained to apply three different rules (namely A, B, C) in consecutive trials. Rules were presented in rapid succession and according to random sequences of triplets (A-A-A, A-B-A, C-B-A). First, a geometric figure (either a circle, a rhombus or a square) appeared in the center of the screen for 1000 ms. Then, a number ranging from 1 to 9 appeared inside the geometric figure and remained on the screen until response or until 3000 ms had elapsed. Based on the geometric figure, participants were therefore asked to decide, as fast and as correct as possible, whether the number was: (A) odd or even, (B) bigger or smaller than five, (C) central (3, 4, 6, 7), or extreme (1, 2, 8, 9). Number 5 was never shown. Two indices of the performance were calculated: the Switch Cost Index and the Backward Inhibition Index. On the one hand, the performance in repetitive trials, in which the rule to apply is the same as the previous one (e.g., A-A-A), is faster than the performance in non-repetitive trials, in which participants switch from one rule to another (A-B-A, C-B-A). This switch cost is therefore interpreted as the time needed for the reconfiguration of the cognitive resources available by the control processes (e.g., Monsell, 2003; Logan, 2003). Specifically, the Switch Cost Index reflects increased mean reaction times on the third trial of switching triplets (A-B-*A*, C-B-*A*), vs. repetition triplets (A-A-*A*). On the other hand, switching back to a recently executed task is harder than switching back to a less recently executed task. The Backward Inhibition Index reflects slower mean reaction times on alternating trials (A-B-A) vs non-alternating trials (C-B-A, A-A-*A*) sequences due to residual inhibition “suffered” by the last A trial of A-B-A sequences compared to the last trial A of C-B-A and A-A-A sequence (Ferlazzo et al., 2014). Higher scores in the Switch Cost Index are interpreted as reflecting less efficient attentional switching capacities, whilst higher scores in the Backward Inhibition Index are interpreted as reflecting more efficient inhibitory capacities (Gratton et al., 2018). All the scores were calculated, excluding trial errors.

### Procedure

The procedure was used in a previous study (Ballesio et al., 2018). Participants were evaluated at the laboratory in the two mornings after a night of habitual sleep and after a night of partial sleep deprivation in their houses. In the sleep deprivation condition, participants were instructed not to go to bed before 1 am and not to wake up after 6 am (5 h of sleep allowed) and to wear the headband at 10 pm in order to verify the wake times. In the habitual night, they were asked to follow their normal sleep and wake times habits, wearing the headband just before going to bed. After both nights, participants were asked to come to the lab to undergo the executive functions assessment. The order of the nights was counterbalanced among participants and between groups in order to control for order effects. Moreover, when the deprivation night occurred first, a night of restoration was guaranteed before the habitual sleep, since a restoration bias might occur after a night of poor sleep (Perlis et al., 2014).

### Data analyses

All data analyses were conducted using the Statistical Package for Social Sciences (SPSS, IBM Corp. 2011) version 26.0. The two EFs indices scores were calculated and re-calculated, excluding trials with errors. Moreover, one subject reported only 11 correct responses after the habitual night and 6 correct responses after the deprivation night, while the mean of the correct responses in both groups was 52 for the HN and 51 for the DN. For this reason, this subject was excluded from the analyses. Since data were normally distributed, we used parametric tests. Group differences in demographic and self-reported measures were tested using Independent sample *t*-tests. Separated mixed design factorial ANOVAs, Group (Binge Eating Group vs. Control Group) × Night (HN vs. DN) were performed on the Total Sleep Time (TST), the number of correct responses, Switch Cost Index and on the Backward Inhibition Index. After that, two further ANCOVAs were separately performed on the two EFs indices inserting BDI-II score as a covariate. This was done since previous literature evidenced poor EFs in depression (Snyder, Miyake & Hankin, 2015). LSD post hoc tests were performed to explore the significant interaction effects. The proportion of type I errors was controlled by setting the False Discovery Rate (FDR) to .05. The FDR was estimated through the procedure described in Storey & Tibshirani (2003). The p0 parameter (Storey, Taylor & Siegmund, 2004) was estimated through the bootstrap procedure (for a general view on the bootstrap procedures, see (Di Nocera & Ferlazzo, 2000). In our results, the .050 level of significance corresponded to an FDR < .050.

## Results

### Group characteristics

Descriptive statistics of the sample and the results of Independent Samples *t*-tests are reported in Table 1. The groups were significantly different for binge eating symptomatology, disordered eating, depression, insomnia severity, and BMI.

**Table 1:** Groups’ characteristics and differences.

	Control group *N* = 13	Binge eating group *N* = 14	***t***(1,25)	***p***
Gender	3 M, 10 F	3 M, 11 F		
Age	24.69 ± 4.13	23.21 ± 3.886	0.96	.347
BMI	20.737 ± 1.277	24.577 ± 5.29	−2.56	**.017**
BES	1.91 ± 1.93	20.36 ± 3.20	−17.93	**<.001**
DEQ	6.85 ± 5.64	39.5 ± 13.64	−8.01	**<.001**
BDI-II	4.87 ± 3.94	15.91 ± 11.26	−3.35	**.003**
ISI	3.58 ± 2.97	7.46 ± 3.71	−2.87	**.009**

**Notes.**

M males F females BMI Body Max Index BES Binge Eating Scale DEQ Disordered Eating Questionnaire BDI-II Beck Depression Inventory-II ISI Insomnia Severity Index

### Sleep manipulation check

Adherence to partial sleep deprivation instructions was inspected by researchers 1 and 2 verifying Zeo recordings of sleep and awake times of each participant and comparing them with subjective data extracted from the sleep diary. Results on TST revealed a significant main effect of the Night (*F*(1, 25) = 130.81, *p* < .001). During the DN both groups slept less (*M*_minutes_ = 274.00 ± 32.53) compared to the Habitual Night (*M*_minute_ = 427.15 ± 57.81 min). No significant Group or Night × Group interactions were found (*p* > .050).

### Results on EFs

A significant Night × Group interaction was found on the Backward Inhibition Index (*F*(1, 25) = 4.68, *p* = .040). LSD post hoc revealed that Bing Eating Group differed from Control Group in the HN (*F*(1, 25) = 5.93, *p* = .020), highlighting higher score of inhibitory control after a night of normal sleep compared to controls. Test comparing the two nights did not evidence significant difference in the Control Group (*F*(1, 25) = 1.91, *p* = .170) and in the Binge Eating Group (*F*(1, 25) = 2.84, *p* = .105).

**Figure 1:**
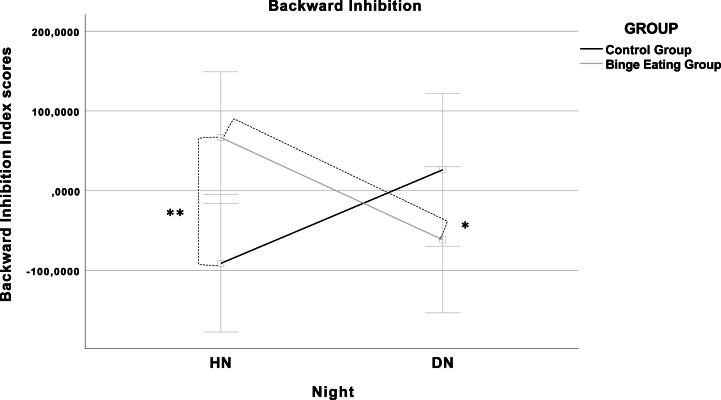
Graphical illustration of the Night × Group interaction (*F*(1, 24) = 6.96, *p* = .014). The covariates in the model are estimated at the following values: Beck Depression Inventory (BDI-II) = 10.59; Error bars: 95% CI; (**) *p* = .020; (*) *p* = .040.

Furthermore, Night × Group interaction on Backward Inhibition remained significant even after inserting depression as covariate (*F*(1, 24) = 6.96, *p* = .014). LSD post hoc showed that the significant difference between Groups in the HN remained (*F*(1, 24) = 6.32, *p* = .019). Moreover, test comparing the two nights revealed a significant difference in the Backward Inhibition in the Binge Eating Group (*F*(1, 24) = 4.69, *p* = .040), and a marginal difference in the Control Group in the opposite direction (*F*(1, 24) = 3.60, *p* = .070). The results of this interaction are shown in Fig. 1. No significant results were found neither on the number of correct responses nor on the Switch Cost Index (*p* > .050).

These results suggested that after the DN the Backward Inhibition was lower in the Binge Eating Group, whereas it was higher in the Control Group, though this difference was not significant. No other results were found on the other variables controlling for BDI-II.

## Discussion

The aim of this study was to compare the effects of a night of partial sleep deprivation on EFs in participants reporting binge eating and healthy controls. Findings revealed that, compared to a habitual night of sleep, participants reporting binge eating showed a smaller Backward Inhibition effect, an index of cognitive inhibition (Couyoumdjian et al., 2010; Gorgoni et al., 2014; Ballesio et al., 2018) after sleep deprivation. This may suggest that at least one cognitive control process included within the EFs plays a potential mediating role in influencing eating behavior when sleep deprived. Moreover, the significant difference in the habitual night between the two groups, with a higher inhibitory control in the binge eating group, seems to be in line with the results by Eneva et al. (2017). Unexpectedly to them, authors found that normal-weight women with BED exhibited higher inhibitory control (*p* < .010) compared to overweight BED, overweight healthy controls, and normal-weight healthy controls. Future investigations are needed to explain these results since they are controversial. In fact, previous studies reported relative EFs weaknesses in inhibitory control in overweight and obese participants with BED (e.g., Mobbs et al., 2011; Svaldi et al., 2014) and an association between increased BMI and poorer executive function test performance (e.g., Gunstad et al., 2007). Nevertheless, other studies found that poorer EFs performance in people with binge eating was no longer significant after controlling for depression (Manasse et al., 2015). Several potential moderators may underly this relationship (e.g., circadian preferences or social facilitation/inhibition, IQ), thus increasing the need for future studies that test models, including different mediators and moderators.

Finally, no main effect of the Group was found, and results on Switch Cost revealed no difference between groups and between nights. The non-significant impact of sleep manipulation on Switch Cost may be explained by the fact that 5 h of sleep may be enough to guarantee an effective attentional switching performance. Supporting this hypothesis, meta-analytic evidence showed that even chronically disturbed sleep, such as that experienced by individuals with chronic insomnia disorder, is associated with an intact (Wardle-Pinkston, Slavish & Taylor, 2019), or even a slight improvement (Ballesio et al., 2018) performance in tasks assessing attentional switching. The lack of difference between groups in the Switch Cost, a measure of cognitive flexibility, is also supported by other evidence suggesting that subjects reporting Binge Eating do not differ for cognitive flexibility from healthy control (e.g., Kittel, Schmidt & Hilbert, 2017; Manasse et al., 2015).

These preliminary findings should be interpreted with caution since this study presents several limitations such as the small and subclinical sample size, as the participants reported symptoms of binge eating and not a clinical diagnose of BED.

## Conclusions

This preliminary study demonstrates that sleep deprivation may affect inhibitory control in participants reporting binge eating, highlighting their potential mediating role in influencing eating behavior. Future studies replicating these preliminary results and investigating the subsequent food intake and eating behaviors are needed to clarify the mediation hypothesis.

##  Supplemental Information

10.7717/peerj.9252/supp-1 Dataset S1 Dataset

## Additional Information and Declarations

### Data Availability

The following information was supplied regarding data availability: The data are available as Supplemental File.
